# The Current Role of Single-Site Robotic Approach in Liver Resection: A Systematic Review

**DOI:** 10.3390/life14070894

**Published:** 2024-07-19

**Authors:** Simone Guadagni, Annalisa Comandatore, Niccolò Furbetta, Gregorio Di Franco, Bianca Bechini, Filippo Vagelli, Niccolò Ramacciotti, Matteo Palmeri, Giulio Di Candio, Elisa Giovannetti, Luca Morelli

**Affiliations:** 1 General Surgery Unit, Department of Translational Research and New Technologies in Medicine and Surgery, University of Pisa, 56126 Pisa, Italy; simone5c@virgilio.it (S.G.); a.comandatore@libero.it (A.C.); niccolo.furbetta@ao-pisa.toscana.it (N.F.); gregorio.difranco@med.unipi.it (G.D.F.); bianca.bechini@gmail.com (B.B.); palmeri.matteo@gmail.com (M.P.); giulio.dicandio@unipi.it (G.D.C.); 2 Department of Medical Oncology, Cancer Center Amsterdam, University Medical Center, 1081 Amsterdam, The Netherlands; 3 Fondazione Pisana per la Scienza, 56017 Pisa, Italy; 4 Endo-CAS (Center for Computer Assisted Surgery), University of Pisa, 56126 Pisa, Italy

**Keywords:** liver surgery, single site, robot, hepatic resection

## Abstract

Background: Liver resection is a critical surgical procedure for treating various hepatic pathologies. Minimally invasive approaches have gradually gained importance, and, in recent years, the introduction of robotic surgery has transformed the surgical landscape, providing potential advantages such as enhanced precision and stable ergonomic vision. Among robotic techniques, the single-site approach has garnered increasing attention due to its potential to minimize surgical trauma and improve cosmetic outcomes. However, the full extent of its utility and efficacy in liver resection has yet to be thoroughly explored. Methods: We conducted a comprehensive systematic review to evaluate the current role of the single-site robotic approach in liver resection. A detailed search of PubMed was performed to identify relevant studies published up to January 2024. Eligible studies were critically appraised, and data concerning surgical outcomes, perioperative parameters, and post-operative complications were extracted and analyzed. Results: Our review synthesizes evidence from six studies, encompassing a total of seven cases undergoing robotic single-site hepatic resection (SSHR) using various versions of the da Vinci© system. Specifically, the procedures included five left lateral segmentectomy, one right hepatectomy, and one caudate lobe resection. We provide a summary of the surgical techniques, indications, selection criteria, and outcomes associated with this approach. Conclusion: The single-site robotic approach represents an option among the minimally invasive approaches in liver surgery. However, although the feasibility has been demonstrated, further studies are needed to elucidate its optimal utilization, long-term outcomes, and comparative effectiveness against the other techniques. This systematic review provides valuable insights into the current state of single-site robotic liver resection and underscores the need for continued research in this rapidly evolving field.

## 1. Introduction

Hepatic resection is a challenging surgical procedure for treating various liver pathologies, both malignant and benign. A thorough understanding of liver surgical anatomy is essential before undertaking hepatic resection [1]. Despite advancements in techniques, devices, and perioperative care, major complications and death can occur following this surgery, and the associated risks should not be underestimated [2]. In this context, minimally invasive options have slowly gained popularity due to challenges such as bleeding control during complex resection and issues with surgical margins, particularly in deeper lesions located in the posterolateral segments [3]. However, with increasing experience in high-volume surgical centers, laparoscopy has emerged as an appealing alternative to open surgery in several hepatic setting, demonstrating similar mid-term results with the benefits of quicker recovery [4,5]. Moreover, in malignant pathologies like hepatocarcinoma or colorectal liver metastasis, overall survival, disease-free survival, and rates of recurrence seem to be comparable to those of traditional surgery [6].

In 2003, the introduction of robotic systems further transformed the surgical landscape, thanks to the inherent advantages, such as 3D stable visualization of the surgical field, endowrist dexterity, and ergonomic console. The da Vinci platform facilitates minimally invasive operations, allowing even less experienced surgeons to perform complex surgical phases that might be challenging laparoscopically. For these reasons, the availability of robotic technology may encourage more surgeons to adopt challenging minimally invasive techniques that they previously might not have considered, such as major hepatectomies [7]. The da Vinci Xi model (Intuitive Surgical, Sunnyvale, CA, USA) overcomes some limitations of the earlier versions with its flexible structure, making it even more suitable for treating bi-lobar lesions or combined surgeries (liver plus primary tumor) in colorectal cancer, without disrupting the workflow [8].

In this context, there has also been a growing emphasis on single-incision surgery, due to its potential cosmetic benefits and reduced post-operative pain, with the first descriptions of this procedure dating back to the early 1990s. From a technical perspective, while laparoscopic single-site surgery primarily struggles with restoring triangulation, the da Vinci robot is equipped with specific tools and software that enable same-sided hand–eye control [9]. Previous versions of single-site robotic surgery lacked endowrist technology; however, Intuitive has recently developed a novel single-site platform, the da Vinci SP, featuring a unique cannula that accommodates three wristed instruments, and a flexible 3D camera. In hepato-biliary surgery, the most commonly described and applied procedure is the single-site cholecystectomy, both laparoscopically and robotically assisted [10]. Various papers and reviews support the feasibility and safety of the robotic option, suggesting that the surgical outcomes may be superior with respect to laparoscopic single-site surgery, except for the cost [11].

Single-site hepatic resection (SSHR) has not developed as rapidly as cholecystectomy, and to date, there is no literature review regarding the role of single-site robotic surgery in this area.

Thus, the aim of the present study is to conduct a systematic review on robotic SSHR and to summarize the current evidence regarding the safety and feasibility of this complex surgery.

## 2. Materials and Methods

We adhered to the guidelines defined in the Preferred Reporting Items for Systematic Reviews and Meta-Analyses (PRISMA) statement for selecting and reporting our articles [12]. Our research was conducted using the PubMed and Scopus databases to identify articles published between January 2009 and January 2024. The search strategy involved various keyword combinations such as “single site robot AND hepatic resection” and “robotic single site AND liver resection”. Additionally, broader search fields like “robotic hepatic surgery” and “robotic single site” were used to ensure the comprehensive retrieval of articles that met the inclusion criteria. We also performed cross-checking of all reference lists to further enhance our analysis.

### 2.1. Study Selection

We initially screened titles and abstracts, followed by a comprehensive assessment of the full texts. Our focus was on manuscripts discussing hepatic resections performed using the robotic single-site approach for all clinical indications. In case series describing multiple operations using the robotic single-site technique, we specifically extracted data on robotic SSHR. Similarly, we only extracted information on the single-site approach in case series also reporting other kinds of hepatic resections. We included all types of hepatic resections: wedge resections, left sectoriectomies, and major hepatectomies. The PICOT (Population, Intervention, Comparison, Outcome, and Time) framework was applied to define our study selection criteria [13]. The following exclusion criteria were applied:

1. Original studies that did not report or from which it was impossible to retrieve perioperative or mid-term outcomes.
2. Review articles, letters, comments, and meta-analyses.
3. Studies in which robotic SSHR was performed in animal models or non-English papers.

### 2.2. Data Extraction

We evaluated in detail the following data: first author, publication year, study type, number of patients treated, reasons for surgical intervention and pre-operative characteristics, age, sex (male/female), body mass index (BMI), type of surgical procedure, single port device used, number and location of the of trocars, length of skin incision, device used for liver transection, length of hospitalization, conversion to open surgery, mortality and morbidity rate (also according to the Clavien–Dindo classification) [14], re-operation rate, pathological results such as diameter and resection margins, and mean follow-up. Any problems related to the surgical intervention during follow-up were also noted.

### 2.3. Quality Assessment and Statistical Analysis

Data collection was performed using Excel software Version 16.86 For quality assessment, we considered study participation, factor measurement, relevance, and applicability. After a comprehensive examination of all qualifying papers, two independent reviewers (SG and LM) critically evaluated data and all results. We calculated the mean value for quantitative data, and we displayed the total number and percentage for qualitative data with a text description in tables when necessary. Centimeters, minutes, and milliliters were used as units of measurement to standardize the presentation. Other measures were converted from millimeters, hours, or liters, whenever necessary. For any missing data, we noted “N.A.–not available” in tables, and these were not considered when calculating any final values. Statistical analyses were conducted using Excel software.

## 3. Results

**Figure 1:**
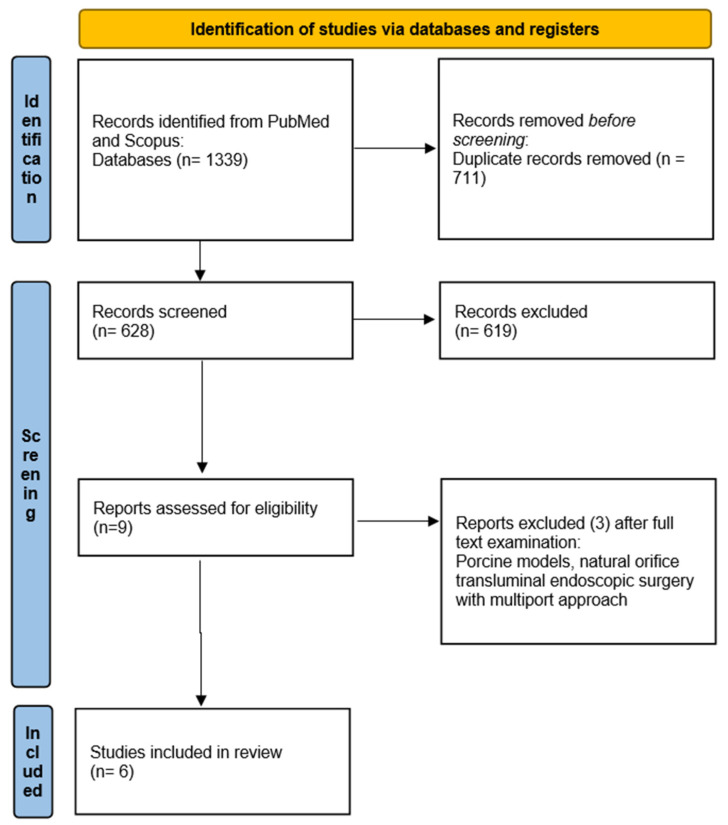
Article selection flow according to PRISMA statement for Reviews.

A thorough examination of the cited databases yielded 1339 articles. After removing duplicates, 628 titles and abstracts were assessed. Following an initial screening, 619 papers were excluded, leaving 9 publications for detailed evaluation. Ultimately, six studies met our inclusion criteria, documenting the outcomes of seven patients (Figure 1). Three articles were excluded for discussing natural orifice transluminal endoscopic surgery with a multiport approach or involving porcine models for eight robotic single-site hepatic procedures. Among the six selected papers included, four are case reports [15,16,17,18] and two [19,20] describe small retrospective single-center experiences. Notably, Kandil et al. [19] detailed seven robotic hepatic resections including two robotic SSHR, while Chong et al. [20] reported three robotic hepato-biliary-pancreatic procedures performed with a single-site approach, with one being a right hepatectomy. The other procedures included were two pancreatic resections. All data are summarized in Table 1 and Table 2.

We retrieved age and gender information from all articles, but BMI data were available from only two [19,20] of six articles, accounting for 33% of the sample. The average age was 51.5 years, and the average BMI was 27.3 kg/m^2^, with the cohort comprising six females and one male. Information about the reasons for surgical intervention were retrieved from all articles. This male patient, reported by Chong et al. [20], was a living right lobe donor, while another patient in Kim et al.’s study [15] was suspected of having Caroli disease affecting segments II–III, with common bile duct stones treated with endoscopic retrograde cholangiopancreatography before surgery. Other cases of the remaining articles [16,17,18,19] involved hepatic resections for lesions detected at second-level imaging: two suspected cases of colorectal liver metastasis, one suspected breast metastasis in segment II, a well-encapsulated tumor in the left lateral sector, and a hemangioma in the caudate lobe.

The articles reported the use of various versions of the da Vinci system: Si in one [19], Xi in two [18,20], and the newest SP version in three [15,16,17]. The average skin incision for single site access was 2.6 cm, ranging from 2.5 cm to 3 cm, all performed in the peri-umbilical area. Four manuscripts described the use of a specific single-site kit manufactured by Intuitive: three with the da Vinci SP Access Port Kit, and one with the da Vinci single-site surgical platform for the Xi. Kandil et al. [19] used an Applied Medical device (Rancho Santa Margarita, CA, USA), and Kim et al. [18] operated in 2020 using a Glove Port (Nelis, Bucheon-si, Gyeonggi-do, Republic of Korea). Most of the authors (66% of total) considered an approach using four robotic trocars. The assistant trocar was inserted into the single-site port in two of seven patients (28.5% of total), in the right upper quadrant for three patients, and in the left lower quadrant for two patients. The surgical interventions included five left lateral segmentectomies, one right hepatectomy, and one caudate lobe resection. An ultrasonic scalpel was used for parenchymal transection in five patients, while fenestrated bipolar plus monopolar forceps were used in two patients. In all left lateral segmentectomies, an endoscopic articulating stapler was used to seal the left hepatic vein, and in 80% of cases, for the Glissonian pedicles. For the right donor hepatectomy, the portal vein, hepatic artery, and bile duct were sealed with Hem-o-lok (Teleflex Incorporated, Co Westmeath, Athlone, Ireland), and the right hepatic vein was sealed with a linear endostapler white cartridge. During the caudate lobe resection [17], only Hem-o-lok was used to clip small feeding branches. The mean operative time was 169.4 min, ranging from 49 to 425 min. Console time reported only by Kandil et al. [19] was 9 and 11 min, respectively, for two cases. Docking time reported by Kim et al. [15] and Liu et al. [16] was 8 min and 14 min, respectively. Specimen extraction was performed throughout the single-site access, except in the cases reported by Chong et al. [20] with a suprapubic incision, and by Kim et al. [18] with a trans-vaginal extraction. The mean estimated blood loss was 69.5 mL, with a range from 10 mL to 300 mL for the right hepatectomy. There were no conversions to multi-port laparoscopy or open surgery. Post-operative pain intensity, measured using the Visual Analogue Scale (VAS), was reported by Liu et al. [16,17] as 3/10 immediately post-operation, and 1/10 on the first post-operative day, while Kim et al. [15] reported a numerical pain score intensity (NIPS) of 3/10 immediately post-operation, and 1/10 on the second post-operative day. Mean hospital stay was 4.2 days, ranging from 2 days (reported by Kandil et al. [19] and by Liu et al. [17]) to 7 days for the right hepatectomy described by Chong et al. [20]. No surgical complications were reported; only one patient described by Kandil et al. [19] experienced a medical issue that slightly prolonged hospitalization without target therapy (grade I according to the Clavien–Dindo classification). In-hospital mortality was 0%.

From a pathological standpoint, the two lesions reported by Kandil et al. [19] were adenoma and colorectal liver metastasis. In the study by Kim et al. [18], the pathological examination confirmed a breast metastasis, while Liu et al. [16] identified an epithelioid angiomyolipoma. The caudate lobe resection [17] confirmed the imaging suspicion of hemangioma. The lesion diameter was reported in three studies with a mean value of 5.1 cm, ranging from 1.4 cm to 7.5 cm. No data were reported on oncologic outcomes, recurrence, and long-term follow-up regarding the development of access site wound infection or incisional hernia. No data were available about cosmesis satisfaction, body image perception, and quality of life after surgery.

## 4. Discussion

The adoption of minimally invasive options for liver surgery has been gradually increasing since its introduction in the early 1990s, particularly when compared to other gastrointestinal procedures. The steep learning curve and the low frequency of liver surgeries in most centers have hindered its widespread use. For a long time, open surgery remained the treatment of choice for all liver pathologies. However, numerous studies by expert surgeons in high-volume centers have highlighted several potential benefits of the minimally invasive approach compared to open liver resection [21]. These advantages include decreased intraoperative blood loss, fewer post-operative complications, reduced need for analgesics, quicker recovery, and shorter post-operative hospital stays [22]. Additionally, in an oncological context, the mid-term outcomes are comparable, and these promising results have fueled a growing interest in minimally invasive liver surgery globally, eventually leading to subsequent guideline meetings on this topic [23]. Today, the minimally invasive approach is considered the gold standard, particularly for metastasectomy and left lateral sectoriectomy, while other complex procedures still require advanced skills and an extensive knowledge of liver anatomy.

In the realm of hepatic surgery, robotic approaches, such as those utilizing the da Vinci robot, represent a significant alternative to traditional laparoscopy. Preliminary studies have shown that complex procedures like major hepatectomies, including extended right, extended left, posterior segments, and living donor hepatectomies, can be successfully performed with this technology [24,25]. The robotic platform enhances various surgical steps, such as the dissection of the hepatic hilum and the hepato-caval plane, hemorrhage control, and performing biliary anastomosis. It also facilitates the integration of advanced technologies like FireFly™ near-infrared fluorescence, which aids in vascular and biliary identification. Dedicated ultrasound probes with Tile-Pro™ functionality simplify the detection of lesions and the assessment of vascular anatomy during parenchymal transection.

In the specific context of colorectal liver metastasis, various studies have underscored the significance of the da Vinci Xi for treating bi-lobar lesions, or in conjunction with primary tumor removal [8,26]. A recent review and meta-analysis by Aboudou et al. [27] found no significant differences in surgical data between robotic and laparoscopic hepatic resection, confirming the safety and efficacy of the robotic approach. Another study on the treatment of colorectal liver metastasis reported higher rates of R0 (complete tumor resection) achievement with robotic surgery. Furthermore, this study highlighted that factors such as high BMI, a history of previous abdominal surgeries, and bi-lobar tumors do not pose barriers to using the latest da Vinci Xi version for these procedures [28].

The shift towards performing surgeries via a single incision is primarily motivated by cosmetic advantages and has gained popularity, especially for cholecystectomy and colorectal procedures. Despite the potential benefits, the interest in single-site hepatic resection (SSHR) has not increased as rapidly as it has for other organs, similar to the adoption trends of standard minimally invasive liver resection. The considerable learning curve associated with laparoscopic single-incision surgery may deter surgeons from adopting this technique [29,30]. However, the development of articulated or double-curved instruments and innovative port devices has made single-incision laparoscopic anatomical hepatectomy both feasible and safe, albeit in a very select group of patients. The challenges include a restricted range of motion and potential conflicts between the operating surgeon and the camera, which can make the procedure time-consuming [31].

In this context, robotic systems, particularly the da Vinci Si and Xi models equipped with specific single-site instruments, have been introduced to improve the ergonomic workflow. An additional innovation is the da Vinci SP, which features three wristed and elbowed instruments and a flexible camera, all delivered through a single 2.5 cm cannula. The initial description of robotic SSHR by Sugimoto et al. [32] in 2011 using a porcine model demonstrated the technical feasibility and safety of this approach. Following this, in 2013, Kandil et al. [19] reported on a series of patients, including two robotic SSHR cases, highlighting benefits such as reduced post-operative pain, shorter hospital stays, and quicker recovery. They also noted that the robotic single-access port minimized instrument crisscrossing and enhanced the utility of the three arms compared to conventional laparoscopy.

More recently, in 2020, a case report detailed a left lateral segmentectomy performed using the da Vinci Xi with a glove single-site port, utilizing natural orifice transluminal surgery for specimen extraction [18]. Additionally, Chong et al. [20] utilized the single-site platform of the da Vinci Xi in three complex hepato-biliary-pancreatic cases, achieving a pancreatoduodenectomy, a combined resection of the common bile duct plus splenic vessels preserving distal pancreatectomy, and a living donor right hepatectomy. Despite longer operative times, the benefits of 3D vision, ergonomic advantages, and reduced internal and external collisions were significant, suggesting that this approach could considerably reduce surgeon stress and fatigue. This experience also highlighted that mainly one endowrist function robotic arm is required for the main procedure, with additional arms used for exposing the operating target, thus not significantly impacting the quality of surgery.

The da Vinci SP^®^ system represents a significant evolution in robotic surgery, moving away from the established multi-port models to a true single-site platform. The intrinsic advantages of this single-port robotic system facilitate various complex operations, and its efficacy has been well documented in the literature for procedures such as nephrectomy, prostatectomy, and gynecologic surgeries [33,34].

During the second study period reviewed, there was a notable increase in the use of the da Vinci SP system, with some case reports published on robotic single-site hepatic resection (SSHR). Liu et al. [16,17] highlighted the safety and efficacy of the da Vinci SP in performing complex surgeries such as left lateral segmentectomy and caudate lobe resection. The latter, a particularly demanding procedure among hepatic resections, showcases the robot’s capabilities in intricate tasks like the fine transection of the dorsal liver plate between the middle and right hepatic veins and the isolation of small portal or hepatic branches directly to the caudate lobe.

Operative times reported in the literature reviewed are quite variable, reflecting the diverse nature of the procedures and the experience levels at different surgical centers. For instance, in 2013, Kandil et al. [19] reported an average operative time of 250 min for their two single-site cases. The duration of surgery can also vary depending on the type of hepatic resection performed; for example, a right hepatectomy performed by Chong et al. [20] took 425 min, compared to a left lateral segmentectomy. Interestingly, the docking times for the da Vinci SP, as shown in two articles [15,16], were much faster (8 and 14 min respectively), underscoring the system’s ease of use and practicality, which confirms the potential of this new single-site platform in reducing setup times and enhancing surgical efficiency.

Impressively, the conversion rate for the procedures reviewed was 0%, with no reports of massive bleeding or other intra-operative accidents, and an average blood loss of 69.5 mL. These data align well with the findings from a recent systematic review on laparoscopic single-site hepatectomy [35], which included 30 cases from the literature involving partial hepatectomy, segmentectomy, or lobectomy, also reporting a 0% conversion rate and a mean blood loss of 50 mL. Such low conversion rates in challenging interventions performed with the da Vinci system are consistent with previous literature and highlight one of the major advantages of robotic assistance: the potential for expanding minimally invasive techniques due to a shorter learning curve.

The specimen was extracted in almost all patients without further incisions, as it is mainly reported to be extracted through the single incision in the umbilical area, except in one case where it was extracted transvaginally. Only in one case, it was extracted via suprapubic incision for a right lobe living donor due to its size. We think that while the first two choices are both in line with the philosophy of a single-incision approach, the need for accessory specimen extraction risks rendering the efforts to perform the entire procedure through a single access pointless.

The post-operative outcomes were generally favorable, with an average hospital stay of 4 days and very few complications. This is in line with several studies on robotic single-site surgery, particularly for cholecystectomy, which have noted good cosmetic results and lower pain intensity using the Visual Analogue Scale (VAS) [36]. Although direct comparisons with laparoscopic approaches in hepatic resection are not available, the pain intensity in the reviewed articles was notably low, rated as 1/10 on post-operative day two, further confirming the reduced invasiveness of this surgical option.

Despite these advantages, robotic single-site hepatic resection (SSHR) has not become as popular as multi-port robotic liver surgery or laparoscopic SSHR. This may be due to the limited number of studies, each with a small sample size, which reflect strict patient-selection criteria driven by safety concerns. These concerns might stem from the absence of an endowrist in the single-site da Vinci Si and Xi platforms and the distance between the operating surgeon and the patient, which can pose challenges in cases of uncontrolled bleeding.

Surprisingly, there was no primary liver cancer among all seven patients reported using SSHR. As we think that this could be a possible indication for SSHR in selected patients, we interpret this absence as a bias because the reported cases appear to be highly selected for the preliminary evaluation of feasibility and efficacy of the single-site robotic hepatic resection technique.

Limitations of the study include possible bias selection of the articles and the low number of procedures performed so far. However, as the literature was lacking a review on this topic, we tried to mitigate this bias by strictly adhering to the PRISMA statement for selecting and reporting our articles and avoiding strong statements.

Finally, although a well-known drawback of the robotic system is related to the high costs, we could not report any data on this point, as currently, there are no cost analyses reported in the literature specifically on single-site hepatic resections.

## 5. Conclusions

The initial feasibility reports from high-volume centers and the subsequent development of the da Vinci SP platform have helped facilitate single-site techniques and simplify the surgical management of liver pathologies. As these technological improvements continue, it is expected that clinical experience with robotic SSHR will accumulate, and its indications will broaden. However, large, randomized studies are needed to fully establish comparisons with standard robotic approaches particularly in terms of safety, cosmesis, and pain relief. Additionally, further research is necessary to determine whether robotic SSHR is safe and feasible for more challenging hepatectomies and the resection of larger malignant tumors. Future studies comparing oncological outcomes in malignant cases to those of standard laparoscopy or open surgery will be crucial in defining the role of this emerging technique.

## Figures and Tables

**Table 1:** Preoperative clinical characteristics of the included patients.

Author.	Year	N of Patient	M/F	Age (Years)	BMI (kg/m^2^)	Pre-Operative Imaging	da Vinci Version
Kim [15]	2021	1	1 F	63	NA	suspected left sector Caroli (removed CBDS with ERCP)	SP
Liu [16]	2022	1	1 F	69	NA	hepatic lesion at CT scan	SP
Liu [17]	2022	1	1 F	30	NA	hemangioma in caudate lobe	SP
Kim [18]	2020	1	1 F	49	NA	lesion identified during follow up for breast cancer	Xi
Kandil [19]	2013	2	2 F	66 ^#^	34.2 ^#^	NA	SI
Chong [20]	2023	1	1 M	32	20.4	liver donor	Xi

Abbreviation: BMI, body mass index; CBDS, common bile duct stones; ERCP, endoscopic retrograde cholangiopancreatography; CT, computer tomography; ^#^ mean value.

**Table 2:** Intraoperative and postoperative outcomes of the included patients.

Author	N of Rob Trocar	AT	Skin Incision (mm)	Type of Intervention	Hepatic Dissection	Operative Time (min)	Docking Time (min)	Blood Loss (mL)	Length of Hospital Stay (Days)	Complications
Kim [15]	4	SS	30	LLS	Bipolar	135	8	50	5	No
Liu [16]	4	RUQ	25	LLS	Harmonic	49	14	10	4	No
Liu [17]	4	RUQ	25	Caudate lobe resection	Harmonic	97	NA	20	2	No
Kim [18]	3	SS	25	LLS	Bipolar	250	NA	15	4	No
Kandil [19]	3	LLQ	30	LLS	Harmonic	60.5 ^#^	NA	22.5 ^#^	3.5 ^#^	Delirium (1 case)
Chong [20]	4	RUQ	25	RH	Harmonic	425	NA	300	7	No

Abbreviation: SS, inside the single-site port; RUQ, right upper quadrant; LLQ, left lower quadrant; RH, right hepatectomy; LLS, left lateral segmentectomy; ^#^ mean value.
